# Stability and Dynamics of the N‑Terminal Domain of TDP-43 and the Effect of Point Mutations

**DOI:** 10.1021/acsomega.5c11735

**Published:** 2026-03-18

**Authors:** Oğuzhan Pınar, Asis K. Jana, Fatih Yaşar

**Affiliations:** † Hacettepe University, Department of Physics Engineering, Ankara 06800, Türki̇ye; ‡ 620070 Sister Nivedita University, Department of Biotechnology, Kolkata 700156, India

## Abstract

TAR DNA-binding protein (TDP-43) has been identified as a major pathological protein in several neurodegenerative diseases. The stable, folded structure of its N-terminal domain (NTD) is thought to be requisite for its physiological function, with any structural disruption potentially leading to aberrant aggregation. In the present study, we have employed long, unbiased all-atom molecular dynamics (MD) simulations to study the conformational stability of the NTD monomer and two of its mutants (L27A and L28A). Since the choice of force field can significantly impact simulation outcomes, we used two different force fields (CHARMM36m and CHARMM36mW). In agreement with experimental data, our simulations using both force fields conclusively demonstrate that the wild-type NTD and the L27A mutant remain structurally stable, while the L28A mutation significantly disrupts tertiary contacts, exposing buried hydrophobic residues and thereby destabilizing the folded structure. Furthermore, across all systems, CHARMM36m simulations exhibited greater overall stability than CHARMM36mW, consistent with our previous study on Aβ fibrils. Importantly, our simulations provide mechanistic insights at the atomic level with significant implications for understanding the NTD’s role in both normal cellular function and pathological aggregation.

## Graphical abstract

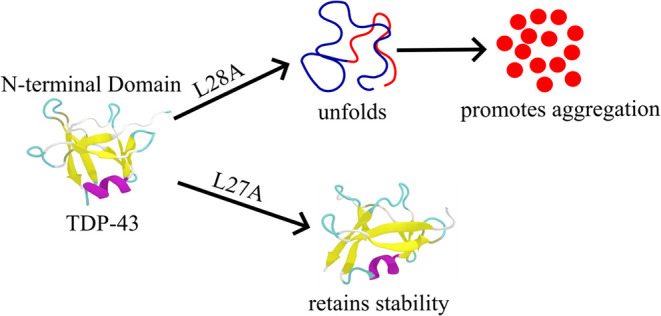

## Introduction

The abnormal accumulation of transactive response DNA-binding protein of 43 kDa (TDP-43) in neurons and glial cells is implicated in several neurodegenerative diseases, including amyotrophic lateral sclerosis (ALS) and frontotemporal lobar degeneration (FTLD).^1,2^ Notably, TDP-43 pathology is also frequently observed in Alzheimer’s disease (AD),^3–5^ the most common cause of dementia worldwide, suggesting that TDP-43 may function as a common pathological substrate across multiple neurodegenerative disorders. TDP-43 is a highly conserved nuclear RNA/DNA-binding protein belonging to the heterogeneous nuclear ribonucleoprotein (hnRNP) family. Under normal physiological conditions, it is primarily localized in the nucleus, where it plays a vital role in the regulation of RNA processing.^6,7^ However, in diseased conditions, TDP-43 becomes mislocalized to the cytoplasm, leading to the formation of cytoplasmic inclusions.^8,9^

Owing to its high aggregation propensity and poor solubility, the complete three-dimensional (3D) structure of TDP-43 remains unresolved to date.^2^ However, structural analyses have identified four major domains: an N-terminal domain (NTD; aa 1–76), two RNA recognition motifs (RRM1: aa 104–176, and RRM2: aa 192–262) connected by a linker region, and a long intrinsically disordered C-terminal domain (CTD; aa 274–414).^10–12^ The CTD contains a hydrophobic conserved region (CR; aa 319–341), a glutamine/asparagine (Q/N)-rich region (aa 345–366), and a glycine-rich region (aa 366–414). Interestingly, the architecture of the CTD closely resembles the prion-like domains found in proteins such as Sup35, Fused in Sarcoma (FUS), and TATA-box binding protein-associated factor 15 (TAF15).^13^ A growing body of experimental evidence reveals the central role of this intrinsically disordered domain in driving pathological aggregation.^1,14,15^ Importantly, a recent study integrating artificial intelligence (AI)-based modeling, computer simulations, and experimental approaches proposed atomic-level structural models of TDP-43 CR multimeric assemblies, revealing crucial insights necessary to understand the early stages of functional TDP-43 CTD assembly.^16^ Notwithstanding the significance of the C-terminal region, recent studies have demonstrated that the N-terminal domain is also essential for efficient aggregation that depletes cells of functional TDP-43.^10,17^ Experimental evidence indicates that under physiological conditions, TDP-43 exists in a dynamic monomer–dimer equilibrium, with dimerization facilitated by NTD.^18,19^ This dimeric form is thought to enhance protein solubility through its role in pre-mRNA splicing and to protect against inclusion formation.^20^ Conversely, other studies suggest that dimerization may facilitate aggregation by increasing the concentration of TDP-43 locally, thereby promoting its pathological aggregation.^18,21^

Experimental studies^22^ by Mompeán et al. demonstrated that NTD must remain stably folded to carry out its physiological functions and maintain predominantly nuclear localization. While the structural integrity of the NTD is essential for proper function, and mutations that disrupt its stability have been shown to impair both dimerization and its ability to regulate mRNA splicing.^22^ In particular, substitution of two highly conserved consecutive Leucine residues at positions 27 and 28 with Alanine revealed distinct functional consequences: the L27A mutation impaired dimerization, while the L28A mutation severely disrupted tertiary contacts and hydrophobic core packing within the NTD, leading to a marked reduction in splicing activity and promoting aberrant localization and aggregation in cells.^22^ Mompeán et al. also reported the solution NMR structure of the NTD in both monomeric and dimeric forms. To date, five NTD structures have been reported, three of which are monomers and two are dimers.^11,20,22–24^ Notably, the overall folds of these reported structures are highly similar, with backbone root-mean-square deviation (RMSD) ranging from 0.5 to 2.5 Å among the monomers.^25^

To get mechanistic insights into the structural dynamics of the NTD at the molecular level, Kumar et al. conducted all-atom molecular dynamics (MD) simulations on the wild-type (WT) and three mutants (L27A, L28A, and V31R).^26^ Using the crystal structure of the NTD dimer (PDB ID: 5MDI)^23^ as the initial structure, they performed simulations over a 250 ns timescale. Simulation results revealed that the mutations disrupted the structural stability and dynamics, with the L28A mutation having the most deleterious effect, consistent with a previous experimental study.^22^ Interestingly, although the simulations showed a significant loss of secondary structure in the L27A mutant, experimental studies reported only minor changes compared to the wild type.^22^ Notably, several studies examining the unfolding pathway of NTD under low pH or elevated temperature in the presence of chemical denaturants have shown that NTD remains well-folded and thermodynamically stable under near-physiological conditions.^27–29^ Importantly, MD simulations have been extensively employed to study IDPs and proteins with intrinsically disordered regions (IDRs), complement experimental observations, and provide atomic-level mechanistic insights that are unattainable using current experimental techniques.^30–37^ The recent advent of AlphaFold has represented a major advance in the predictions of protein 3D structures. Building on this advance, several IDP simulation studies have employed AlphaFold-generated structural models as initial configurations for proteins/regions lacking experimentally resolved structure, providing valuable insights into the conformational dynamics of IDPs and their assemblies.^16,37^

Note that previous experimental study has indicated the on-pathway presence of monomer during TDP-43 aggregation.^38^ As recently reported by Doke et al., the role of TDP-43 monomer during aggregation remains poorly understood, plausibly due to limited information on the factors governing the stabilization of TDP-43 monomer.^1^ As noted earlier, several experimental studies have characterized the folded structure of the N-terminal domain of TDP-43 monomer^11,20,22^ and further indicate that stably folded NTD monomer promotes dimerization, which is pertinent to both its physiological function and pathological aggregation.^22^ In this study, we carried out microsecond-scale, fully atomistic, and unbiased MD simulations on the experimentally resolved NTD monomeric structure^11^ and its L27A and L28A mutants to elucidate the underlying molecular mechanisms governing the stabilization of NTD monomer. Since the choice of force field can significantly influence the results of MD simulations, particularly for IDPs, due to the complexity of their energy landscapes, numerous studies have demonstrated the impact of force fields on the structures, dynamics, and thermodynamics of amyloid-forming proteins.^39–41^ Accordingly, we have employed two different force fields, CHARMM36m and CHARMM36mW, to assess force field dependence.^42^ Notably, CHARMM36mW is also based on CHARMM36m but includes more favorable protein–water dispersion interactions without altering water properties.^42^ This reoptimization of protein–water interactions reduces the overcompaction of IDPs and has been shown in multiple studies to yield improved agreement with experimental data.^41,42^ In consistent with experimental results, our simulations using both force fields demonstrate that the wild-type NTD and the L27A mutant retain structural stability, whereas the L28A mutation results in a pronounced loss of stability. However, this destabilizing effect of the L28A mutation is considerably less pronounced in the CHARMM36m force field. Interestingly, across all systems, regardless of wild-type or mutants, CHARMM36m simulations exhibited greater overall stability compared to CHARMM36mW, agreement with our previous study on Aβ fibrils.^43^

## Materials and Methods

### Simulation Set-Up

The solution NMR structure of the N-terminal domain (NTD) of TDP-43 (PDB entry: 2N4P),^11^ consisting of 77 residues, was the starting structure of our study and is shown in detail in Figure 1. This structure includes six β-strands and one α-helix, arranged as β1 (I5–V7)-β2 (I16–I18)-α (L28–Q34)-β3 (G40–R44)-β4 (R55–V57)-β5 (I60–H62)-β6 (V72–N76). The structure was mutated at positions 27 and 28 to Alanine using Visual Molecular Dynamics (VMD)^44^ to generate the L27A and L28A mutants, respectively. In all systems, the N- and C-terminal residues were capped with NH_3_^+^ and COO^–^ groups, respectively. Before simulation, each system was placed in a periodic TIP3P^45^ water box with a minimum distance of 15 Å between any protein atom and the box edge. Systems were neutralized by adding Na^+^ and Cl^–^ ions, and additional ions were further added to achieve a physiological ion concentration of 150 mM NaCl.

**Figure 1:**
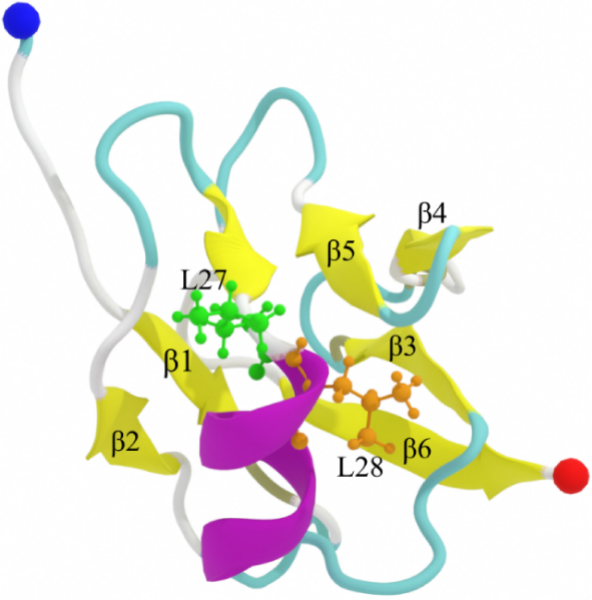
Solution NMR structure of N-terminal domain of TDP-43 (PDB ID: 2N4P).^11^ The β-strands and the α-helix are colored yellow and violet, respectively. Residues 27 and 28 are displayed in CPK representation and colored green and orange, respectively. The N- and C-terminal residues are indicated by blue and red spheres, respectively.

### Simulation Protocols

Simulations were conducted using the GROMACS 2022.2 simulation package.^46^ Each system was first energy-minimized using the steepest descent algorithm for 50,000 steps to remove any steric clashes. Following minimization, the systems were equilibrated first under NVT conditions at 310 K for 100 ps, followed by another 100 ps NPT equilibrations at 310 K and 1 atm. During equilibrations, positional restraints with a force constant of 1000 kJ mol^–1^ nm^–2^ were imposed to the heavy atoms of the protein, allowing the surrounding water molecules to equilibrate around the solute. Production simulations were then carried out under NPT conditions (310 K, 1 atm) using a 2 fs time step. Three independent production runs of 3.0 μs each, starting from different initial velocities, were conducted for each system. Temperature was maintained at 310 K via the v-rescale thermostat^47^ (coupling constant: 0.1 ps), and pressure was regulated at 1 atm using the Parrinello–Rahman barostat^48^ (coupling constant: 5 ps). Water molecules are kept rigid using the SETTLE algorithm, and the LINCS algorithm^49^ was applied to constrain nonwater covalent bonds involving hydrogen atoms. Long-range electrostatic interactions were calculated using the particle-mesh Ewald (PME) method with a cutoff distance of 12 Å.^50^ For short-range Lennard-Jones interactions, the same 12 Å cutoff was applied, with smoothing starting at 10.5 Å.

### Simulation Analyses

Simulation trajectories were analyzed using GROMACS tools, VMD,^44^ and our in-house scripts, and visualized with both VMD^44^ and PyMOL software.^51^ Root-mean-square deviation (RMSD) and root-mean-square fluctuation (RMSF) for all backbone atoms in the NTD were computed relative to the experimentally resolved solution NMR structure using the GROMACS tools gmx rms and gmx rmsf, respectively. Prior to RMSD and RMSF calculations, the translational and rotational superposition of the protein with respect to the initial structure is performed using least-squares fitting. The radius of gyration of the protein was computed using the GROMACS tool gmx gyrate, and secondary structure analysis was performed via the Dictionary of Secondary Structure in Proteins (DSSP) method^52^ implemented in the GROMACS do_dssp tool. Additionally, the radial distribution function of water oxygen atoms around the protein C_α_ atoms was computed using the GROMACS tool gmx rdf. Using VMD, we calculated the total number of intrapeptide hydrogen bonds and residue–residue contact maps. Contacts were defined by a 7.0 Å cutoff for the minimum distance between heavy atoms within a residue pair, and hydrogen bonds were identified with a donor–acceptor distance cutoff of 3.0 Å and a donor–hydrogen–acceptor angle greater than 160°. Solvent-accessible surface area (SASA) was estimated in VMD^44^ using a spherical probe with a radius of 1.4 Å. To identify and analyze large-scale, functionally relevant motions, we performed principal component analysis (PCA).^53^ For PCA analysis, the covariance matrix of atomic positional fluctuations was constructed from the mass-weighted Cartesian coordinates of C_α_ atoms, using the GROMACS tool gmx covar. The covariance matrix was constructed using the following equation:

$$C_{ij}=\langle \left(x_{i}-\left\langle x_{i}\right\rangle \right)\left(x_{j}\right)-\left\langle x_{j}\right\rangle \rangle$$

Here, *x*_1_,···,*x*_*3N*_ represent the mass-weighted Cartesian coordinates of the *N* C_α_ atoms, and ⟨···⟩ denotes averaging over all trajectory snapshots. Diagonalization of the covariance matrix was then performed using the GROMACS tool gmx anaeig to obtain eigenvectors (principal components, PCs) and their corresponding eigenvalues. Each PC represents a direction of motion within the system, with the eigenvalue reflecting the amplitude of fluctuation along that direction. The PCs with the largest eigenvalues represent the dominant collective motions of the system. Note that, in most systems, only a few PCs are sufficient to capture the majority of motions within the system, as well as functionally relevant dynamics. We computed the free-energy surface in PC space from the probability density of the distribution along the first two PCs using the following equation:

$$\Delta G=-k_{B}T\ln\left[\frac{p}{p_{max}}\right]$$

Here, *k*_B_ is Boltzmann’s constant, *T* is the absolute temperature, *p* is the probability derived from the distribution of the first two PCs, and *p*_max_ is the corresponding maximum probability. Note that we first discussed the results obtained from CHARMM36mW simulations, and the CHARMM36m simulation results are presented and compared with the CHARMM36mW simulations in the “Impact of Force Field” section.

## Results and Discussions

### Effect of Mutation on Global Conformational Properties

We begin our analysis by computing root-mean-square-deviation (RMSD), a key metric in MD simulation commonly used to evaluate structural stability and conformational changes of systems. We have computed RMSD for the backbone atoms of the protein across all systems, relative to the solution NMR structure,^11^ following alignment to it. Figure 2 presents the time evolution of RMSD, averaged over three independent trajectories per system; corresponding data for each trajectory are shown in Figure S1 in the Supporting Information. In both WT and L27A systems, RMSD remains nearly constant throughout the simulation after initial fluctuations, indicating structural stability. In contrast, the L28A system exhibits a significant increase in RMSD in all three trajectories after 1.5 μs, suggesting substantial structural changes. Notably, no significant difference in RMSD values was observed between the WT and L27A systems. The mean RMSD, averaged over the final 1.0 μs across the three trajectories, was 2.6 ± 0.2 Å for WT, 2.7 ± 0.3 Å for L27A, and notably higher at 5.0 ± 0.9 Å for L28A. Furthermore, we have computed the RMSD based on the per-residue heavy-atom contact distance relative to the initial structure as a function of simulation time for all systems; this metric is referred to as cRMSD. Figure S2 in the Supporting Information shows the time evolution of cRMSD averaged over three independent trajectories for each system; corresponding data for each trajectory are provided in Figure S3 in the Supporting Information. Importantly, the overall pattern of the cRMSD data closely matches that observed in the conventional RMSD analysis. Consistent with the RMSD data, both the WT and L27A systems exhibit nearly constant cRMSD values after initial fluctuations, indicating that both systems reach equilibrium at an early stage of the simulation. In contrast, the L28A system shows a pronounced increase in cRMSD, in agreement with the RMSD data, indicating conformational changes upon L28A mutation. No significant changes in cRMSD value are observed after 2.0 μs. In addition, we have computed the fraction of native contacts, with the corresponding results shown in Figure S4 of the Supporting Information, which also display a similar trend. For the contact analysis, a cutoff distance of 4.5 Å was used, and contacts were considered only for residue pairs separated by at least two residues. Notably, no significant changes in the fraction of native contacts are observed in the L28A system after 2.0 μs. Based on these findings, the last 1.0 μs of trajectories were used for all systems in subsequent analyses, including PCA, *R*_g_, RMSF, and others.

**Figure 2:**
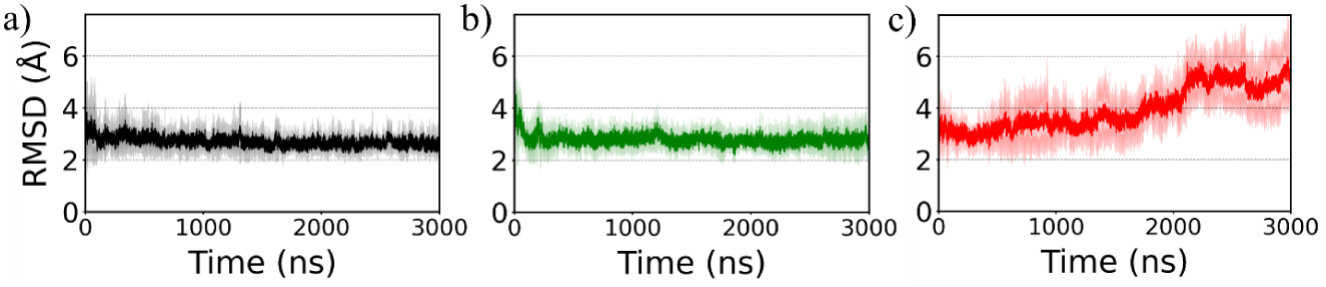
Time evolution of backbone RMSD for the (a) WT (black), (b) L27A (green), and (c) L28A (red) systems. Data are averaged over three independent trajectories per system, with the shaded region representing the standard deviation.

To further characterize global conformational properties, we next computed the radius of gyration (*R*_g_), the solvent-accessible surface area (SASA) of hydrophobic residues, and the total number of intramolecular hydrogen bonds of the protein across the simulation trajectories for all systems. In Figure 3, we compared the distributions of *R*_g_, hydrophobic SASA, and hydrogen bond counts among the three systems. Notably, both the *R*_g_ and hydrophobic SASA distributions are shifted toward higher values in the L28A system, indicating a more expanded and solvent-exposed conformation. The average *R*_g_ values, calculated over the final 1.0 μs across all three trajectories, were 11.8 ± 0.1 Å for WT, 11.9 ± 0.1 Å for L27A, and 12.3 ± 0.2 Å for L28A. The corresponding mean SASA values for the hydrophobic residues are 1593.0 ± 93.3 Å^2^ for WT, 1643.2 ± 136.4 Å^2^ for L27A, and 1825.6 ± 157.9 Å^2^ for L28A. Thus, *R*_g_ and hydrophobic SASA increase by about 4.2 and 14.6% in L28A system. Notably, previous experimental study^11^ indicates that NTD stability is maintained by burial of numerous nonpolar residues, and further identified I^5^, G^24^, A^63^, W^68^, Y^43^, Y^73^, R^6^, V^7^, I^16^, I^18^, S^20^, T^25^, V^26^, T^30^, V^31^, F^35^, A^38^, G^40^, L^41^, N^45^, G^53^, V^54^, G^59^, I^60^, L^61^, G^67^, L^71^, V^72^, and V^75^ as predominantly buried. We computed the SASA values of these residues for all systems along the simulation trajectories. The average SASA values, measured over the final 1.0 μs for all three trajectories, were 789.0 ± 102.3 Å^2^ for WT, 863.8 ± 101.9 Å^2^ for L27A, and 1182.3 ± 176.1 Å^2^ for L28A. The L28A mutation leads to approximately a 50% increase in solvent exposure of these buried residues, while the increase in the L27A system is comparatively minor at about 9.4%. Consistent with experimental observations,^22^ our simulations further reveal that the hydrophobic side chain of L27 is largely surface-exposed. The average SASA of L27 calculated over the final 1.0 μs across all three trajectories, was 78.2 ± 15.6 Å^2^ for the WT system. Note that, surface-exposed and evolutionarily conserved hydrophobic residues are known to commonly form binding surfaces.^54,55^ Since experimental study has identified L27 as a key residue at the dimerization interface,^22^ therefore, the L27A mutation impairs dimerization. Furthermore, the distribution of total number of hydrogen bonds (H-bonds) within the protein shows reduction of H-bonds in the L28A system, decreasing by about 16.3% in the L28A system. Overall, these analyses provide compelling evidence that the L28A mutation shifts the conformational ensembles of NTD monomers toward extended, more solvent-exposed, and loosely packed conformations. In contrast, the L27A mutation does not significantly alter the conformational properties of the NTD monomer relative to WT.

**Figure 3:**
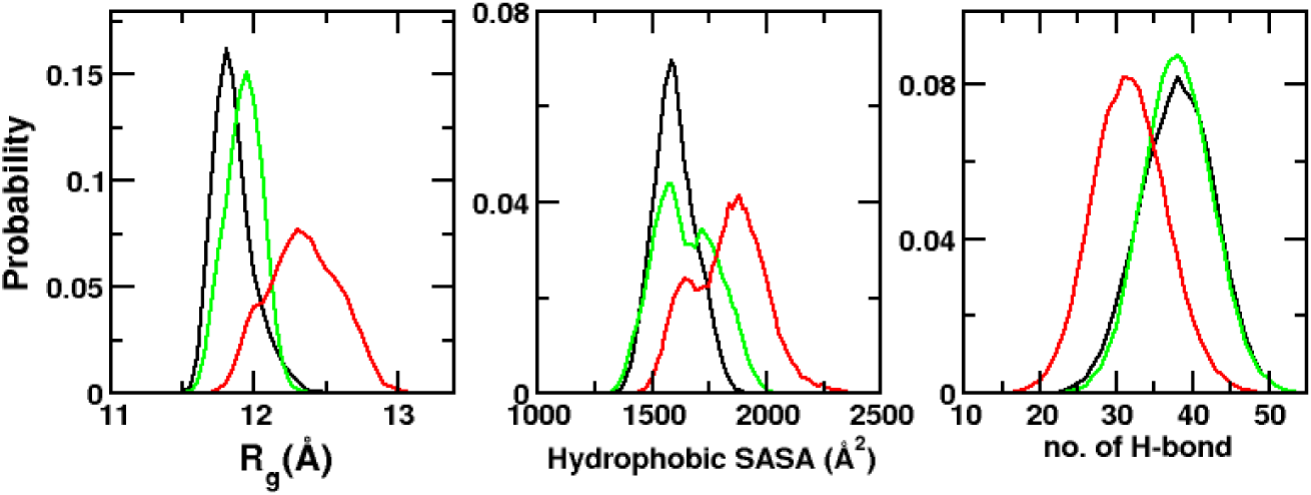
Normalized distributions of radius of gyration (*R*_g_), solvent-accessible surface area of hydrophobic residues (Hydrophobic SASA), and the number of hydrogen bonds (H-bond) for the WT (black), L27A (green), and L28A (red) systems. Data are averaged over the final 1.0 μs of three independent trajectories, for each system.

### Effect of Mutation on Local Structural Dynamics

To quantify the impact of the mutation on local structural dynamics, we computed the residue-wise backbone RMSF along the simulation trajectories, with the results shown in Figure S5 in the Supporting Information. The L28A variant exhibits significantly increased flexibility compared to the WT and L27A systems, except in the region comprising residues 46 and 47. No significant differences were observed between the WT and L27A systems overall, although the L27A system displays slightly higher flexibility than WT, except in the region spanning residues 21 to 25. Notably, this is consistent with previous experimental findings, where NMR relaxation analysis revealed increased dynamics in the L28A variant on the fast picosecond–nanosecond timescale.^22^

We next calculated the secondary structural propensities for all systems using the DSSP protocol. As seen in Figure 1, the α-helix content is much lower than the β-sheet. The percentages of α-helix and β-sheet for each system obtained from the simulations are summarized in the Supporting Information (Table S1). In the WT system, the β-sheet content is about 36%, which decreases slightly to 33% in the L27A system. In contrast, the L28A system shows a marked reduction to 24%, indicating substantial disruption of the secondary structure. This is also clearly seen in Figure 4, where we have shown the representative final configuration extracted from simulations of WT, L27A, and L28A systems. A similar trend is observed for α-helices, with 9% in WT, 8% in L27A, and a further decrease to 5% in L28A. To get further details, we also computed residue-wise β-sheet and α-helix propensities; corresponding data for each system are provided in the Supporting Information (Tables S2 and S3). In the native structure, the β-strands are positioned at β1 (residues 5–7), β2 (residues 16–18), β3 (residues 40–44), β4 (residues 55–57), β5 (residues 60–62), and β6 (residues 72–76), while the α-helix spans residues 28–34. Analysis of the per-residue propensities confirms the presence of β-strand/α-helix within these regions over the simulations. Moreover, extra β-strand segments emerged at residues 25–27 and 50–54 in all systems during the simulations. Note that, only residues with α-helix/β-sheet occurrence greater than 10% over the simulations were considered as part of the respective structural regions. β-strands are widened for all systems. Overall, β-strands are widened across systems. However, occurrences of both α-helix and β-sheet are markedly reduced in the L28A system compared to WT and L27A, due to the emergence of irregular turns and bends. These observations conclusively demonstrate that the L28A mutation significantly destabilizes the NTD monomer.

**Figure 4:**
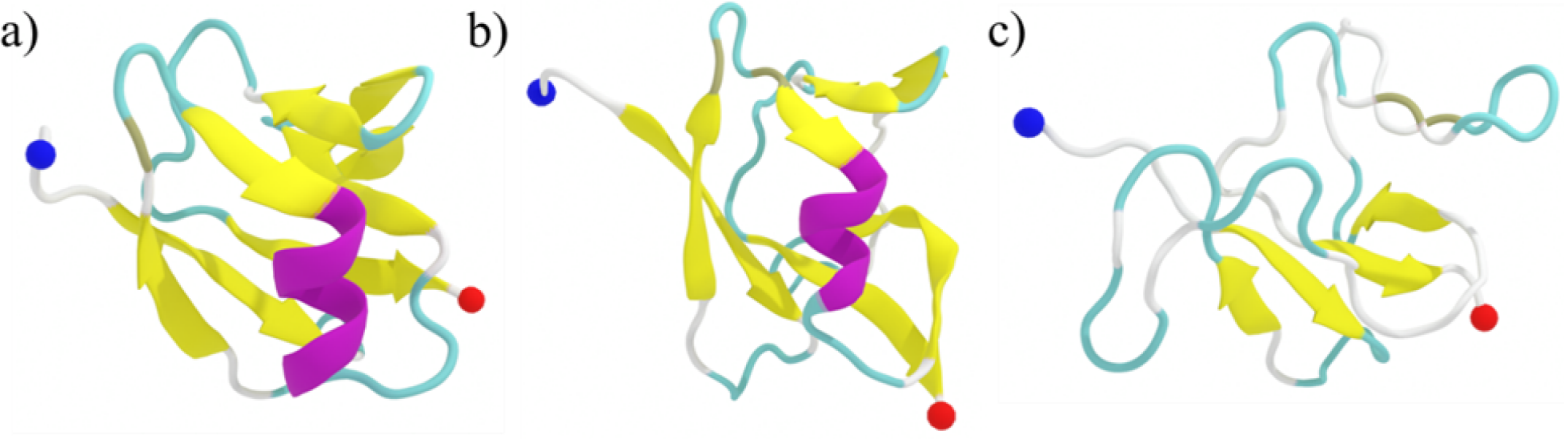
Representative final configurations (at 3.0 μs) from simulations of the (a) WT, (b) L27A, and (c) L28A systems. The β-strands and the α-helix are colored yellow and violet, respectively. The N- and C-terminal residues are indicated by blue and red spheres, respectively.

### Loss of Tertiary Contacts upon Mutation

To obtain a more detailed understanding of NTD unfolding upon mutation, we compared the tertiary structures of the WT, L27A, and L28A mutants. Figure 5 presents the residue–residue contact probabilities for all systems, and the corresponding differences upon mutation are shown in Figure S6 in the Supporting Information. Analysis of the contact patterns reveals a significant loss of nonlocal native contacts and the emergence of transient interactions in the L28A mutant. In particular, long-range contacts and hydrogen bonds between residues 1–7 and 16–24, including interactions between β1 and β2 involving residue pairs I^5^–I^16^, I^5^–I^18^, R^6^-E^17^, and V^7^–I^16^ are significantly disrupted in the L28A mutant. In contrast, new long-range contacts emerge between residues 1–16 and 60–73. In the L27A mutant, we also observe the formation of long-range contacts between residues 5–16 and 69–73, including backbone hydrogen bonds involving R^6^-L^71^, T^8^-Y^73^, and T^8^-L^71^. Moreover, the L28A mutant shows an increased frequency of contacts between residues 5–13 and β3, involving I^5^–Y^43^, T^8^-R^44^, D^10^-R^44^, E^11^-N^45^, and D^13^-R^44^.

**Figure 5:**
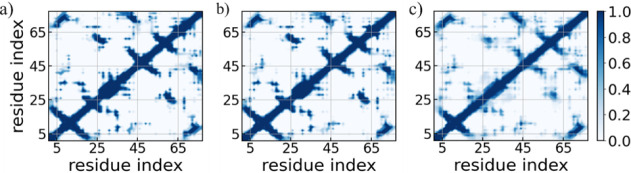
Residue–residue contact probabilities of the (a) WT, (b) L27A, and (c) L28A systems. Data are averaged over the final 1.0 μs of three independent trajectories, for each system.

Medium-range hydrogen bonds within the turn region (E^21^–T^25^) connecting β2 and the α-helix involving residue pairs E^21^-T^25^ and D^22^-T^25^ are substantially lost, thereby destabilizing this region. Further disruption is observed in long-range contacts between this turn and residues 61–68 involving residue pairs D^23^-H^62^, G^24^-A^63^, G^24^-W^68^, and T^25^-L^61^. The L28A mutation also leads to the loss of backbone hydrogen bonds between β4 and β5 involving residue pairs R^55^–H^62^ and V^57^–I^60^, resulting in a substantial disruption of β-sheet arrangement in this region. Moreover, the L28A mutation also results in the loss of numerous long-range hydrophobic contacts involving the mutation site and nearby residues. These include residue pairs V^26^-L^41^, V^26^–V^54^, V^26^-L^56^, V^26^–I^60^, V^26^-A^63^, V^26^–W^68^, L^27^-L^61^, A^28^-A^38^, A^28^-L^41^, A^28^-L^56^, V^31^-A^38^, V^31^-L^41^, and V^31^–V^75^. A further loss of long-range hydrophobic and π–π stacking interactions is observed between the turn region (F^35^-A^38^) and β6, involving residue pairs F^35^–V^75^, F^35^–Y^77^, A^38^-V^75^, and A^38^-Y^77^. In addition, key backbone hydrogen bonds and π–π stacking contacts between β3 and β6, involving residue pairs G^40^-N^76^, R^42^-V^74^, and Y^43^–V^73^, are also disrupted in the L28A mutant. Collectively, the loss of these stabilizing interactions exposes buried hydrophobic residues, contributing significantly to the overall destabilization observed in the L28A mutant. Alternatively, a slightly higher propensity of contact formation is observed between residues 28–38 and 54–62 in the L28A mutant, involving key contacts such as A^28^-V^57^, V^31^-L^56^, A^33^-L^61^, F^35^-L^56^, F^35^-L^61^, and A^38^-L^61^. Notably, changes in per-residue contact probabilities in the L27A mutant relative to WT are minimal (see Figure S6 in the Supporting Information), indicating that the L27A variant largely retains the native fold, in close agreement with a previous experimental study.^22^

### Essential Dynamics

We have employed principal component analysis (PCA) to determine the essential dynamics present in the simulation and to examine the impact of mutations on these motions. We have used the final 1.0 μs of three independent trajectories for all systems for PCA analysis. The first three principal components captured 86%, 84%, and 85% of the total motion for WT, L27A, and L28A, respectively. The individual contributions of these principal components for each system are detailed in Table S4 of the Supporting Information. Figure 6 presents the free-energy landscape projected onto the first two principal components (PC1 and PC2). We performed cluster analysis to get representative structures from the lowest free-energy regions, labeled 1 to 7 in Figure 6. Representative structures are shown around the side of Figure 6. Furthermore, *R*_g_, hydrophobic SASA, total percentage of α-helix, and β-sheet content for these lowest free-energy regions are presented in Table S5 of the Supporting Information. Notably, all systems exhibit two well-defined clusters. The WT samples a relatively narrow conformational space, ranging from 0.2 to 3.0 nm along PC1 and −1.0 to +2.5 nm along PC2. The L27A mutant explores a slightly broader space (−3.1 to −0.7 nm along PC1 and −1.5 to +2.5 nm along PC2), while the L28A mutant displays the largest conformational space, spanning −5.0 to −1.4 nm on PC1 and −4.2 to +3.4 nm on PC2. In the WT free-energy landscape, two well-separated energy basins are observed: a deeper one centered around conformation 2 and a shallower one around conformation 1. Conformation 2 (RMSD = 2.4 Å) is slightly more stable than conformation 1 (RMSD = 2.5 Å); however, both represent near-native or folded states. Similarly, the L27A system exhibits two distinct energy minima, corresponding to conformations 3 and 4, with RMSD values of 2.6 Å and 2.5 Å, respectively. The L28A system displays two broad and spread-out basins. The representative conformations show much higher RMSD values (4.1 Å for conformation 5, 5.7 Å for 6, and 5.6 Å for 7), indicating substantial structural deviations and large-scale conformational rearrangements due to the L28A mutation. Overall, essential dynamics analysis conclusively demonstrates that the L28A mutant samples a significantly larger conformational space than the WT and L27A systems. The L28A mutation increases collective motions and can destabilize NTD.

**Figure 6:**
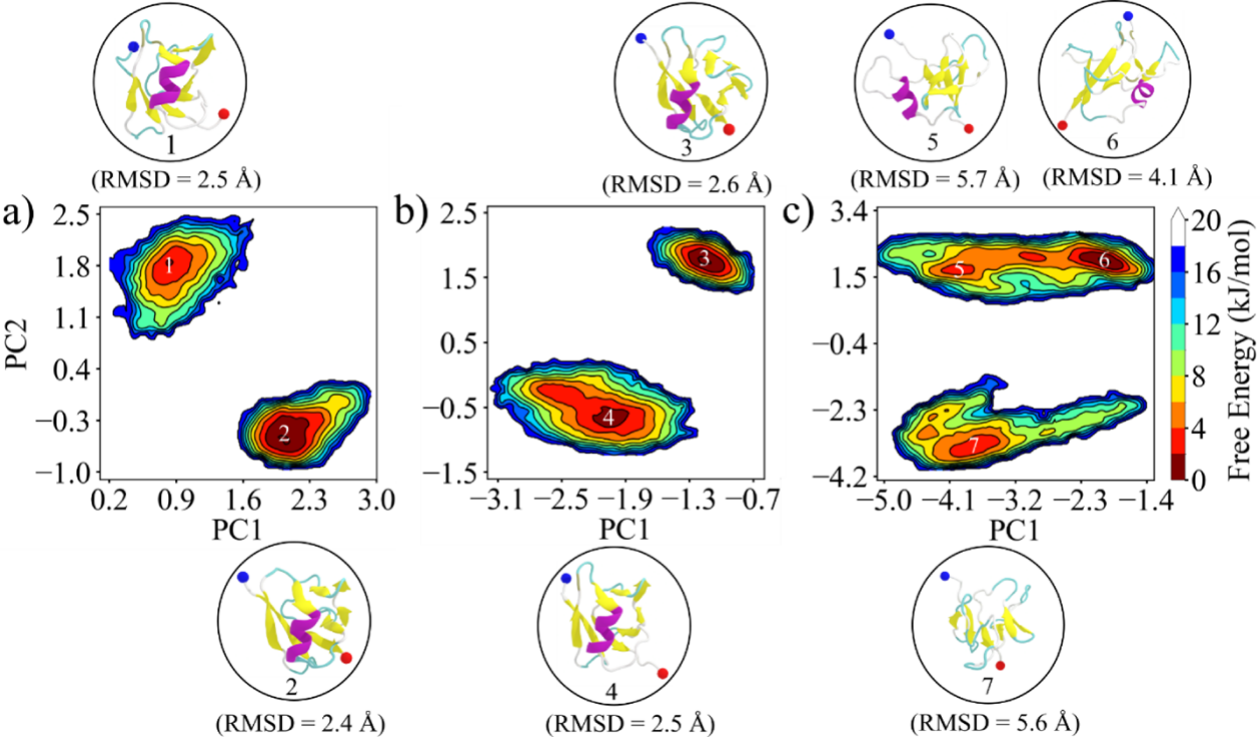
Two-dimensional free-energy landscapes as functions of the first (PC1) and second (PC2) principal components for (a) WT, (b) L27A, and (c) L28A systems. The lowest-energy structure corresponding to each minima is shown along with its backbone RMSD value. β-strands and the α-helix are colored yellow and violet, respectively, while the N- and C-terminal residues are marked with blue and red spheres, respectively.

### Local Hydration Properties around Wild-Type and Mutant NTDs

It is well-known that water molecules around a protein or polypeptide significantly influence its folding and conformational dynamics. They also play a vital role in the pathological aggregation of IDPs.^56,57^ In light of this, we have examined the properties of water around the wild-type and mutant NTDs. Figure S7 in the Supporting Information presents the radial distribution function, g­(r), of water oxygen atoms around the C_α_ atoms of the amino acid residues. An increase in the height of the first solvation peak is observed for the L28A mutants, indicating enhanced solvent accessibility of the NTD monomer upon mutation. This likely arises from disruption of tertiary contacts by the L28A mutation, leading to exposure of residues that are buried in the wild-type NTD. A marginal increase in the first solvation peak relative to the WT is also observed for the L27A mutants. Notably, the radial distribution function analysis correlates well with the SASA results discussed in the previous section. Overall, our findings indicate that conformational changes in the NTD monomer strongly influence the local arrangement of surrounding water molecules. Future studies from our group will investigate in detail water dynamics around TDP-43 and its mutants to further elucidate their role in conformational transitions and aggregation process.

### Impact of Force Field

For force field comparison, we conducted three independent 3 μs trajectories for each system using the CHARMM36m force field. Representative final configurations of all systems obtained from the CHARMM36m simulations are presented in Figure S8 in the Supporting Information. The wild-type NTD and L27A mutant exhibits similar stability, with a mean RMSD of 2.5 Å averaged over the final 1.0 μs of three independent trajectories. The time evolution of RMSDs from these simulations is shown in Figures S9 and S10 in the Supporting Information. In agreement with the CHARMM36mW results, the L28A mutant displayed higher RMSD values relative to WT and L27A mutant; however, the value was substantially lower than that observed in the CHARMM36mW simulations (see Table S6 in the Supporting Information). The mean RMSD, averaged over the final 1.0 μs over the three trajectories, was 3.3 ± 0.5 Å for CHARMM36m and 5.0 ± 0.9 Å for CHARMM36mW force field. We next compared the *R*_g_, hydrophobic SASA, and the total number of intramolecular hydrogen bonds within the protein among the three systems. The distributions of *R*_g_, hydrophobic SASA, and hydrogen bond count are shown in Figure S11 in the Supporting Information. Notably, in contrast to the CHARMM36mW simulations, both *R*_g_ and hydrophobic SASA were slightly lower in the L27A mutant relative to WT, suggesting its marginally enhanced structural stability. Averaged over the final 1.0 μs and three independent trajectories, the *R*_g_ values were 11.8 ± 0.1 Å for WT and 11.7 ± 0.1 Å for L27A, while the mean hydrophobic SASA was 1534.2 ± 84.2 Å^2^ for WT and 1506.6 ± 63.7 Å^2^ for the L27A mutant. Similar to the CHARMM36mW simulations, the L28A mutant displayed increased *R*_g_ and hydrophobic SASA relative to both WT and L27A, though the extent of the increase was lower than that observed in CHARMM36mW simulations (see Table S6 in the Supporting Information). For instance, the mean hydrophobic SASA for L28A mutant obtained from CHARMM36m simulations was 1719.6 ± 95.8 Å^2^, corresponding to a 12.1% increase in hydrophobic surface exposure relative to WT, compared to a 14.6% increase in CHARMM36mW. Likewise, the L28A mutation led to a 15.3% reduction in intramolecular hydrogen bonds relative to WT, less than the 16.1% decrease observed in CHARMM36mW. In contrast, the L27A mutant showed a modest increase in hydrogen bonding compared to WT, further supporting its slightly enhanced stability, as also reflected in the *R*_g_ and hydrophobic SASA trends.

To further assess the effects of mutation at local scale, we calculated residue-wise backbone RMSF and secondary structural propensities for all systems. The residue-wise backbone RMSF profiles are presented in Figure S12 in the Supporting Information, and the total percentages of α-helix and β-sheet content are also summarized in the Supporting Information (Table S7). Residue-wise percentages of α-helix and β-sheet occurrences are also listed in the Supporting Information (Tables S8 and S9). The L28A mutant exhibited increased flexibility compared to the WT and L27A mutants, although the extent of this increase was notably smaller than that observed in the CHARMM36mW simulations, particularly in the region spanning residues 18–40. Consistent with the *R*_g_, hydrophobic SASA, and H-bond count analyses, the L27A mutant showed slightly reduced flexibility relative to WT, suggesting marginally enhanced structural stability. In line with this, the L27A mutant showed higher β-sheet content compared to WT, whereas the L28A mutant exhibited a substantial reduction in β-sheet content relative to both WT and L27A (see Table S7 in the Supporting Information). Overall, simulations with both force fields consistently indicate that the L28A mutation leads to a substantial loss of structural stability, though the effect is less pronounced in the CHARMM36m simulations. Notably, CHARMM36m simulations suggest marginally higher structural stability in the L27A mutant compared to WT, in contrast to the results obtained with CHARMM36mW. Interestingly, across all systems, irrespective of wild-type or mutants, CHARMM36m simulations yielded lower RMSD, RMSF, and reduced hydrophobic SASA, and higher H-bond counts relative to CHARMM36mW, indicating greater overall stability in CHARMM36m force field. This is consistent with our previous study on Aβ fibrils.^43^

## Conclusions

Emerging studies demonstrate the key role of the N-terminal domain (NTD) in the pathological aggregation of TDP-43, and the consequent loss of its physiological function.^21,58^ Under physiological conditions, TDP-43 exists in a monomer–dimer equilibrium.^18,19^ Solution NMR studies^11,20^ show that the NTD is stably folded, which is pertinent to its physiological role and pathological aggregation. Notably, prior mutagenesis studies have shown that substituting two highly conserved consecutive Leucine residues at positions 27 and 28 with Alanine leads to distinct functional consequences.^22^ The L27A mutation retains stable folding and can regulate mRNA splicing but impairs dimerization, whereas the L28A mutation destabilizes the NTD fold, leading to a significant reduction in splicing activity and promoting aberrant localization and aggregation in cells. Therefore, studying the conformational dynamics of the NTD is essential, as even subtle perturbations such as those introduced by point mutations can disrupt the balance between functional dimerization and pathological aggregation. In the present study, we comparatively examined the structural dynamics and stability of the NTD monomeric structure and its L27A and L28A mutants using fully atomistic MD simulations on the microsecond time-scale. The conformational ensemble sampled during MD simulations is strongly influenced by the choice of force field, particularly for proteins in the monomeric state.^41,59^ Therefore, we employed two different force fields, CHARMM36m and CHARMM36mW, to evaluate their impact on the conformational behavior of the NTD. Note that, replica-exchange MD (REMD) simulations have been widely used for IDPs and other proteins to enhance conformational sampling;^60–63^ however, we did not conduct REMD because our unbiased atomistic MD simulations on the microsecond timescale show close agreement with previous experimental results.^22^

Our simulation analyses, including RMSD, *R*_g_, SASA, and H-bond, clearly indicate that the L27A mutation preserves the structural integrity of the NTD monomer and does not significantly alter its overall conformational properties relative to the WT. In contrast, the L28A mutation shifts the conformational ensemble of the NTD toward more extended, solvent-exposed, and loosely packed conformations. Secondary structure analysis further revealed that the L28A mutant exhibits a reduced α-helical and β-sheet content compared to WT and L27A mutant. The free energy landscape (FEL) derived from PCA analysis demonstrated that WT and L27A mutant adopt well-defined energy minima separated by high-energy barriers, reflecting conformational stability. Conversely, the L28A mutant exhibits a broader and shallower energy landscape, indicative of greater conformational flexibility that allows the protein to adopt multiple distinct conformations. Furthermore, we employed the MUpro,^64^ SAAFEC,^65^ and DynaMut^66,67^ servers to predict the effects of single-point mutations on the stability and dynamics of the NTD monomer. In agreement with our MD simulations and previous experimental findings,^22^ all three servers consistently indicate that the L28A mutation exerts the maximum destabilizing effect (see Table S10 in the Supporting Information).

While the previous simulation study^26^ qualitatively agrees with prior experimental observations,^22^ the simulations were short (∼250 ns) and limited to a single trajectory per system. Such short simulations are sufficient for system equilibration and for capturing fast, local motions such as bond vibrations and side chain rearrangements, they remain strongly influenced by the initial configuration, resulting in limited conformational sampling. In the present study, we try to overcome these limitations by conducting unbiased MD simulations using two different force fields on the 3.0 μs timescale and by generating three independent trajectories for each system. This substantially enhances conformational sampling, reduces bias arising from the initial structure and force field choice, and enables a statistically reliable assessment of the structural stability and dynamics.

Importantly, simulations using both force fields consistently demonstrate that the wild-type NTD and L27A mutant remain structurally stable over the simulation, whereas the L28A mutation significantly disrupts the NTD fold. Notably, the destabilizing effect of the L28A mutation is less pronounced in CHARMM36m simulations. Across all systems, CHARMM36m simulations exhibit greater structural stability and reduced conformational flexibility, consistent with our previous findings on Aβ fibrils.^43^ Surprisingly, CHARMM36m simulations show slightly enhanced stability for the L27A mutant relative to the WT, which is not in agreement with CHARMM36mW simulations and prior experimental observations.^22^ The primary difference between these two force fields is the refinement of protein–water interactions by increasing the Lennard-Jones parameters of hydrogen in the TIP3P water model.^42^ Our computations clearly show that protein–water interactions have a significant impact on the conformational flexibility of the peptide systems. Therefore, maintaining an appropriate balance between the force field and the water model remains essential for accurately sampling conformational space, particularly in studies involving highly dynamic systems such as IDPs. Future studies from our group will investigate this effect in more detail across various IDPs using enhanced sampling techniques.

## Supplementary Material



## Data Availability

All methodological details, including the PDB code and simulation conditions, are provided in the Materials and Methods section. The simulation software and force fields used in this study are openly accessible, as are the visualization software and trajectory analysis tools.

## Notes

Time evolution of backbone RMSD, cRMSD, and fraction of native contacts across independent trajectories; residue-wise backbone RMSF; total percentages of α-helix and β-sheet content, and residue-wise α-helix (%) and β-sheet (%) obtained from simulations; differences in per-residue contact frequencies upon mutations; individual contribution of the first three PCs to the total variance; *R*_g_, hydrophobic SASA, and total percentage of α-helix and β-sheet content for the lowest free-energy regions identified by PCA analysis; radial distribution functions of water oxygen atoms around C_α_ atoms; representative final configurations from CHARMM36m simulations; time evolution of the average backbone RMSD and of backbone RMSD for independent trajectories from CHARMM36m simulations; mean values of RMSD, *R*_g_, hydrophobic SASA, and H-bond count obtained from CHARMM36m and CHARMM36mW simulations; normalized distributions of *R*_g_, hydrophobic SASA, and H-bond counts from CHARMM36m simulations; residue-wise backbone RMSF from CHARMM36m simulations; total percentages of α-helix and β-sheet content, and residue-wise α-helix (%) and β-sheet (%) obtained from CHARMM36m simulations; prediction of stability changes upon point mutations using the MUpro, SAAFEC, and DynaMut servers (PDF)
