# Supplementary material for: Stability and Dynamics of the N‑Terminal Domain of TDP-43 and the Effect of Point Mutations
Source: ACS Omega. 2026 Mar 18;11(12):19124–33. doi: 10.1021/acsomega.5c11735 (PMC13044679; doi:10.1021/acsomega.5c11735)
Supplement: Supplementary file 1 [file ao5c11735_si_001.pdf]

Supporting Information

Stability and Dynamics of the N-terminal Domain of TDP-43 and the effect of Point Mutations

Oğuzhan Pınar*[a], Asis K. Jana*[,b] and Fatih Yaşar[*,a]

[a]Hacettepe University, Department of Physics Engineering, Ankara 06800, TÜRKİYE

[b]Sister Nivedita University, Department of Biotechnology, Kolkata, INDIA

*Corresponding Authors: fatih@hacettepe.edu.tr

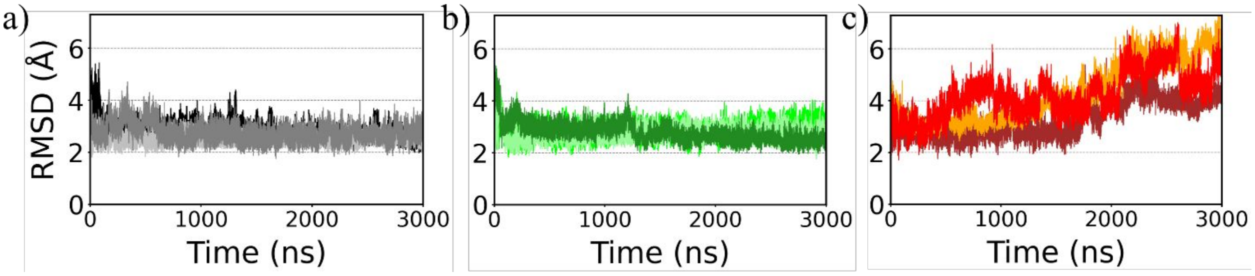

Figure S1: Backbone RMSD as a function of simulation time for three independent trajectories of the (a) WT, (b) L27A, and (c) L28A systems.

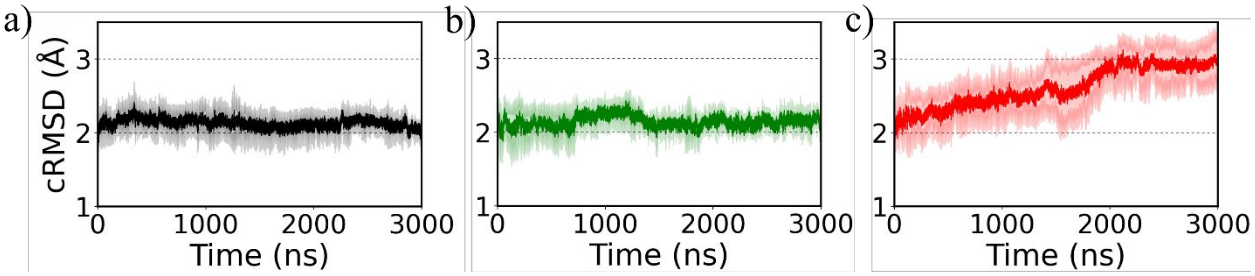

Figure S2: RMSD of the per-residue heavy-atom contact distance (cRMSD), relative to the initial structure as a function of simulation time for the (a) WT, (b) L27A, and (c) L28A systems. Data are averaged over three independent trajectories per system, with the shaded region representing the standard deviation.

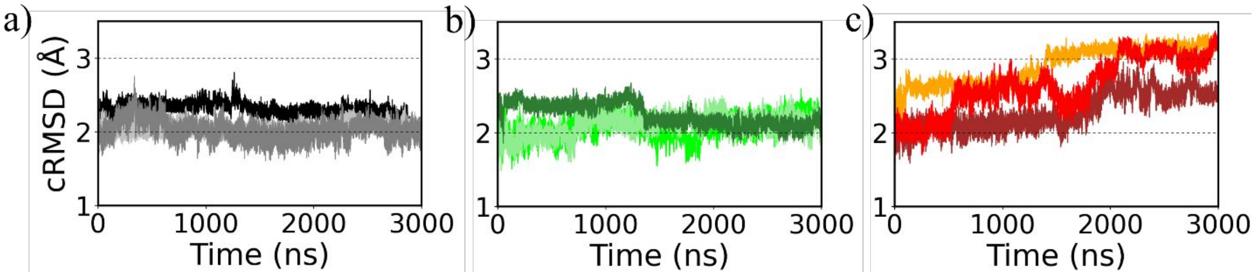

Figure S3: RMSD of the per-residue heavy-atom contact distance (cRMSD), relative to the initial structure, as a function of simulation time for three independent trajectories of the (a) WT, (b) L27A, and (c) L28A systems.

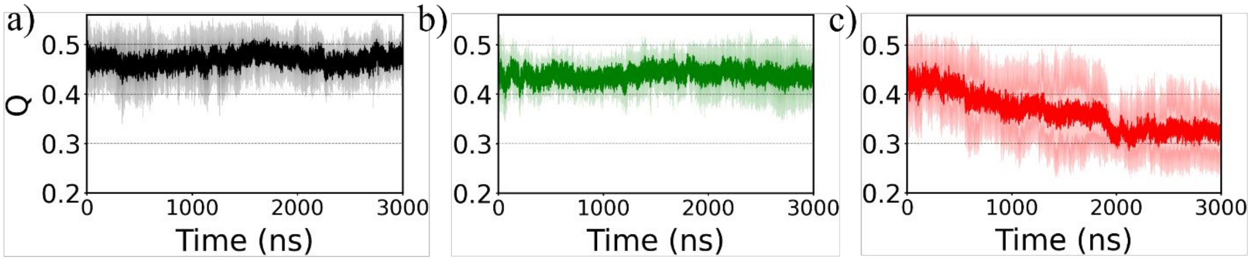

Figure S4: Fraction of native contacts (Q) over simulation time for the (a) WT, (b) L27A, and (c) L28A systems. Data are averaged over three independent trajectories per system, with the shaded region representing the standard deviation.

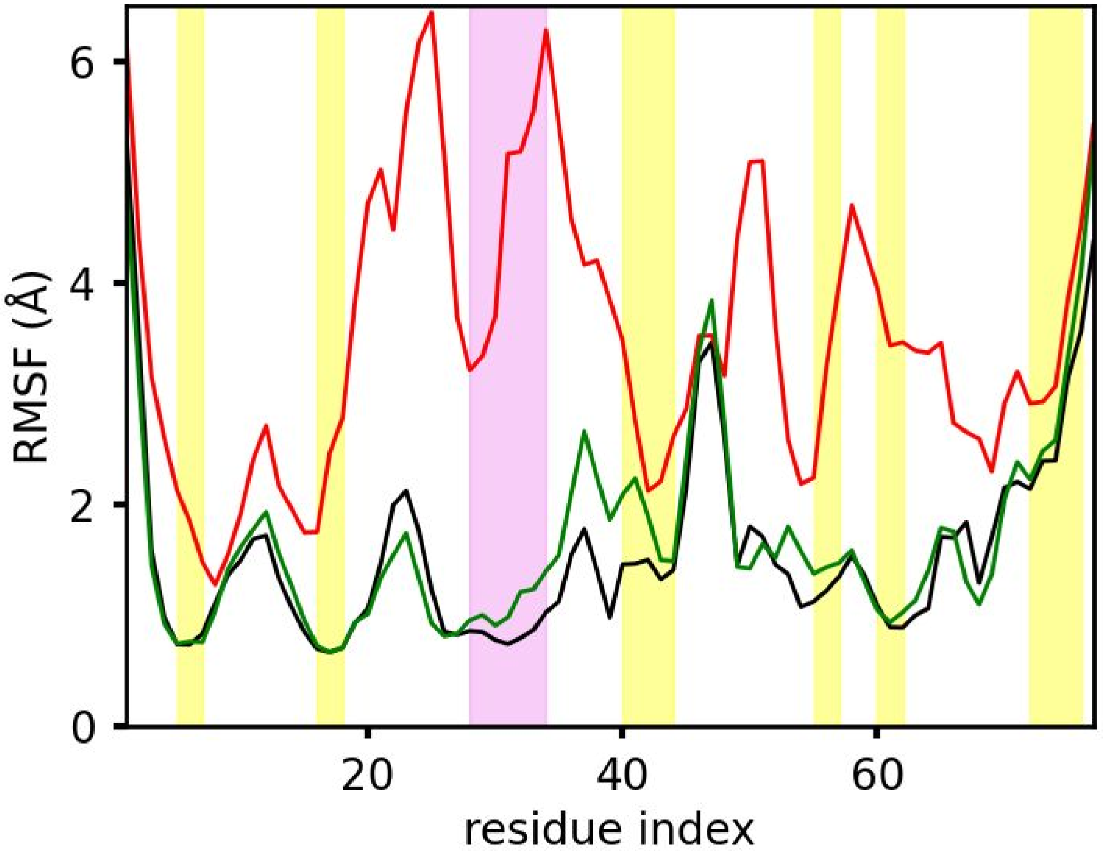

Figure S5: Residue-wise backbone RMSF for WT (black), L27A (green), and L28A (red) systems. Data are averaged over the final 1.0 μs of three independent trajectories per system. β-sheet and α-helix regions in the native structure are colored in yellow and violet, respectively.

Table S1: Total percentages of α-helix and β-sheet content measured from simulations of the WT, L27A, and L28A systems. Data were averaged over the final 1.0 μs of three independent trajectories per system.

System	α-helix	β-sheet
WT	9.0	36.0
L27A	8.0	33.0
L28A	5.0	24.0

Table S2: Residue-wise β-sheet (%) computed over the final 1.0 μs of three independent trajectories per system. β-strand regions present in the native structure are indicated, with the corresponding β-sheet (%) from the simulations shown in bold. Mutation sites are shown in red, and residues with β-sheet occurrence over 10% during the simulation, along with their corresponding β-sheet (%), are shown in green.

	residue	WT	L27A	L28A
	1 MET	0.0	0.0	0.0
	2 SER	1.3	2.4	1.9
	3 GLU	15.4	16.4	2.8
	4 TYR	78.1	74.8	26.9
**β1**	**5 ILE**	**97.7**	**99.8**	**34.9**
**β1**	**6 ARG**	**99.9**	**100.0**	**74.4**
**β1**	**7 VAL**	**100.0**	**100.0**	**99.5**
	8 THR	94.9	98.8	98.0
	9 GLU	0.0	0.0	0.0
	10 ASP	0.0	0.0	0.0
	11 GLU	0.0	0.0	0.0
	12 ASN	0.0	0.0	0.0
	13 ASP	0.0	0.0	0.0
	14 GLU	0.0	0.0	0.0
	15 PRO	0.5	0.3	0.3
**β2**	**16 ILE**	**99.9**	**99.9**	**64.0**
**β2**	**17 GLU**	**99.9**	**99.9**	**63.8**
**β2**	**18 ILE**	**97.1**	**99.8**	**33.3**
	19 PRO	78.1	74.8	24.1
	20 SER	16.5	16.9	0.1
	21 GLU	1.7	0.7	0.1
	22 ASP	0.1	0.0	0.0
	23 ASP	0.0	0.0	0.0
	24 GLY	0.0	0.0	0.0
	25 THR	51.1	40.9	13.6
	26 VAL	96.0	98.4	15.9
	27 LEU	95.8	98.3	15.9
	28 LEU	0.0	0.0	0.1
	29 SER	0.0	0.0	0.0
	30 THR	0.0	0.0	0.0
	31 VAL	0.0	0.0	0.0
	32 THR	0.0	0.0	0.0

	33 ALA	0.0	0.0	0.0
	34 GLN	0.0	0.0	0.0
	35 PHE	0.0	0.0	0.0
	36 PRO	0.0	0.0	0.0
	37 GLY	0.0	0.0	0.0
	38 ALA	0.0	16.6	0.2
	39 CYS	0.0	17.0	1.1
**β3**	**40 GLY**	**40.4**	**26.3**	**15.4**
**β3**	**41 LEU**	**66.5**	**38.2**	**81.5**
**β3**	**42 ARG**	**92.8**	**62.6**	**94.6**
**β3**	**43 TYR**	**100.0**	**86.0**	**97.5**
**β3**	**44 ARG**	**99.9**	**81.6**	**63.8**
	45 ASN	16.7	5.5	3.0
	46 PRO	0.0	0.0	0.0
	47 VAL	0.0	0.0	0.0
	48 SER	0.0	0.3	3.0
	49 GLN	0.0	0.3	3.0
	50 CYS	16.7	5.5	3.0
	51 MET	97.1	40.8	9.0
	52 ARG	96.4	59.4	15.3
	53 GLY	92.2	54.9	8.1
	54 VAL	0.8	0.6	0.0
**β4**	**55 ARG**	**71.4**	**63.2**	**62.2**
**β4**	**56 LEU**	**99.4**	**98.7**	**62.8**
**β4**	**57 VAL**	**98.2**	**97.5**	**60.8**
	58 GLU	0.0	0.0	0.0
	59 GLY	0.0	0.0	0.0
**β5**	**60 ILE**	**99.9**	**99.8**	**76.6**
**β5**	**61 LEU**	**100.0**	**99.9**	**78.7**
**β5**	**62 HIS**	**84.1**	**73.7**	**75.8**
	63 ALA	0.0	0.0	1.9
	64 PRO	0.0	0.0	1.9
	65 ASP	0.0	0.0	1.9
	66 ALA	0.0	0.0	1.9
	67 GLY	0.0	0.0	2.8
	68 TRP	0.0	0.0	0.9
	69 GLY	0.8	0.1	1.5
	70 ASN	6.9	25.9	62.4
	71 LEU	28.2	65.7	66.3
**β6**	**72 VAL**	**73.1**	**84.6**	**94.5**
**β6**	**73 TYR**	**96.1**	**79.6**	**97.7**
**β6**	**74 VAL**	**67.8**	**44.4**	**64.3**
**β6**	**75 VAL**	**66.6**	**50.6**	**49.7**
**β6**	**76 ASN**	**40.4**	**38.5**	**12.6**
	77 TYR	0.0	0.0	0.0

Table S3: Residue-wise α-helix (%) computed over the final 1.0 μs of three independent trajectories per system. α-helix regions present in the native structure are indicated, with the corresponding α-helix (%) from the simulations shown in bold. Mutation sites are highlighted in red.

	residue	WT	L27A	L28A
	1MET	0.0	0.0	0.0
	2 SER	0.0	0.0	0.0
	3 GLU	0.0	0.0	0.0
	4 TYR	0.0	0.0	0.0
	5 ILE	0.0	0.0	0.0
	6 ARG	0.0	0.0	0.0
	7 VAL	0.0	0.0	0.0
	8 THR	0.0	0.0	0.0
	9 GLU	0.0	0.0	0.0
	10 ASP	0.0	0.0	0.0
	11 GLU	0.0	0.0	0.0
	12 ASN	0.0	0.0	0.0
	13 ASP	0.0	0.0	0.0
	14 GLU	0.0	0.0	0.0
	15 PRO	0.0	0.0	0.0
	16 ILE	0.0	0.0	0.0
	17 GLU	0.0	0.0	0.0
	18 ILE	0.0	0.0	0.0
	19 PRO	0.0	0.0	0.0
	20 SER	0.0	0.0	0.0
	21 GLU	0.2	0.1	0.1
	22 ASP	0.2	0.1	0.1
	23 ASP	0.2	0.1	0.1
	24 GLY	0.2	0.1	0.1
	25 THR	0.0	0.0	0.0
	26 VAL	0.0	0.0	0.0
**α-helix**	**27 LEU**	**0.0**	**0.0**	**0.0**
**α-helix**	**28 LEU**	**99.8**	**99.7**	**65.0**
**α-helix**	**29 SER**	**100.0**	**99.9**	**65.5**
**α-helix**	**30 THR**	**100.0**	**99.9**	**65.6**
**α-helix**	**31 VAL**	**100.0**	**99.9**	**65.7**
**α-helix**	**32 THR**	**98.5**	**97.0**	**55.9**
**α-helix**	**33 ALA**	**89.4**	**76.2**	**31.5**
**α-helix**	**34 GLN**	**88.1**	**69.1**	**24.3**
**α-helix**	**35 PHE**	**0.0**	**0.0**	**0.0**
	36 PRO	0.0	0.0	0.0
	37 GLY	0.0	0.0	1.3
	38 ALA	0.0	0.0	1.3
	39 CYS	0.0	0.0	1.3
	40 GLY	0.0	0.0	1.3
	41 LEU	0.0	0.0	0.0
	42 ARG	0.0	0.0	0.0
	43 TYR	0.0	0.0	0.0
	44 ARG	0.0	0.0	0.0
	45 ASN	0.0	0.0	0.0
	46 PRO	0.0	0.0	0.0

	47 VAL	0.0	0.0	0.0
	48 SER	0.0	0.0	0.0
	49 GLN	0.0	0.0	0.0
	50 CYS	0.0	0.0	0.0
	51 MET	0.0	0.0	0.0
	52 ARG	0.0	0.0	0.0
	53 GLY	0.0	0.0	0.0
	54 VAL	0.0	0.0	0.0
	55ARG	0.0	0.0	0.0
	56 LEU	0.0	0.0	0.0
	57 VAL	0.0	0.0	0.0
	58 GLU	0.0	0.0	0.0
	59 GLY	0.0	0.0	0.0
	60 ILE	0.0	0.0	0.0
	61 LEU	0.0	0.0	0.0
	62 HIS	0.0	0.0	0.0
	63 ALA	0.0	0.0	0.0
	64 PRO	0.0	0.0	0.0
	65 ASP	0.1	0.0	0.0
	66 ALA	0.1	0.0	0.0
	67 GLY	0.3	0.0	0.0
	68 TRP	0.3	0.0	0.0
	69 GLY	0.2	0.0	0.0
	70 ASN	0.2	0.0	0.0
	71 LEU	0.0	0.0	0.0
	72 VAL	0.0	0.0	0.0
	73 TYR	0.0	0.0	0.0
	74 VAL	0.0	0.0	0.0
	75 VAL	0.0	0.0	0.0
	76 ASN	0.0	0.0	0.0
	77 TYR	0.0	0.0	0.0

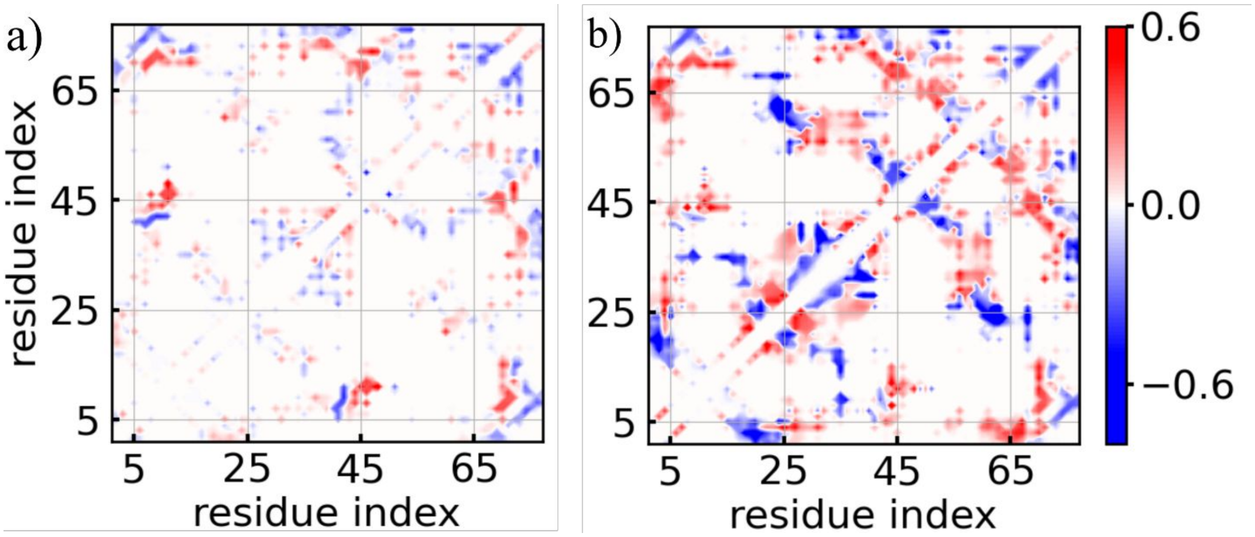

Figure S6: Differences in per-residue contact frequencies upon (a) L27A and (b) L28A mutation. Data are averaged over the final 1.0 μs of three independent trajectories per system.

Table S4: Individual contribution of the first three PCs to the total variance, derived from PCA analysis.

System	Principal Component	Variance (%)
WT	PC1	58.86
	PC2	21.60
	PC3	5.44
L27A	PC1	55.43
	PC2	21.00
	PC3	6.90
L28A	PC1	48.42
	PC2	28.06
	PC3	8.77

Table S5: R_g, hydrophobic SASA, and the total percentages of α-helix and β-sheet content for the lowest free-energy regions, labeled 1 to 7 in Figure 6.

System		R_g (Å)	Hydrophobic SASA ($Å^2$)	α-helix	β-sheet
WT	1	11.9	1644.5	9.0	33.0
	2	11.8	1541.7	9.0	38.0
L27A	3	11.8	1525.3	8.0	38.0
	4	11.9	1702.5	9.0	30.0
L28A	5	12.3	1870.4	8.0	22.0
	6	12.0	1665.3	7.0	22.0
	7	12.5	1864.8	0.0	28.0

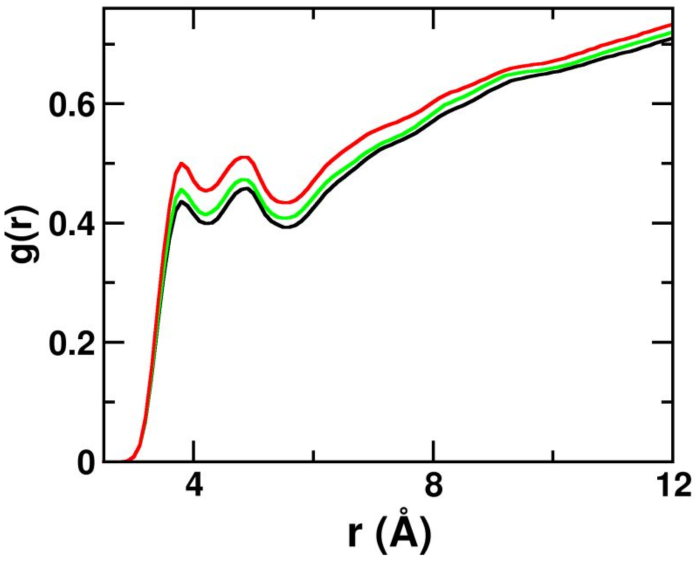

Figure S7: Radial distribution functions of water oxygen atoms around the C_α atoms of amino acid residues for the WT (black), L27A (green), and L28A (red) systems. Data are averaged over the final 1.0 μs of three independent trajectories per system.

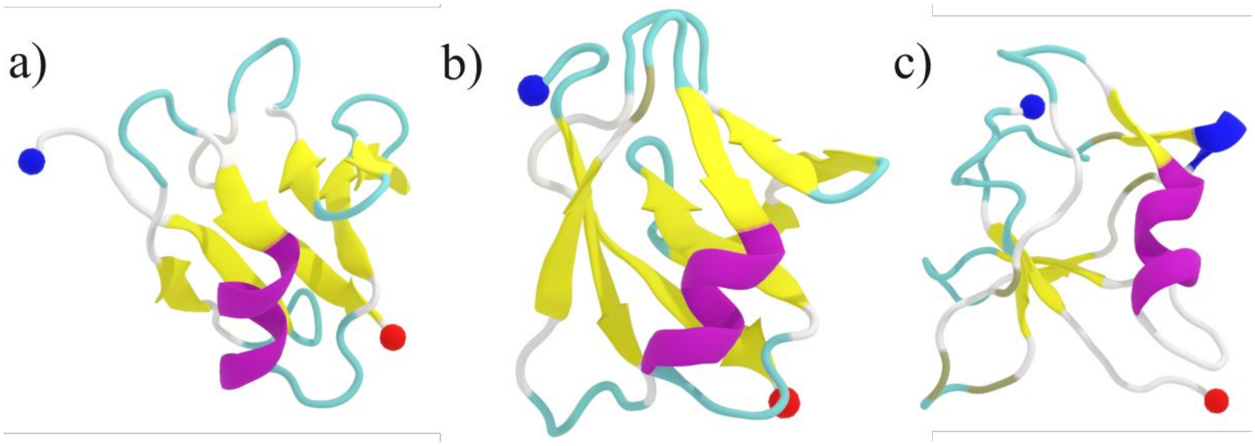

Figure S8: Representative final configurations (at 3.0 μs) from CHARMM36m simulations of the (a) WT, (b) L27A, and (c) L28A systems. β-strands and the α-helix are colored yellow and violet, respectively, while the N- and C-terminal residues are marked with blue and red spheres, respectively.

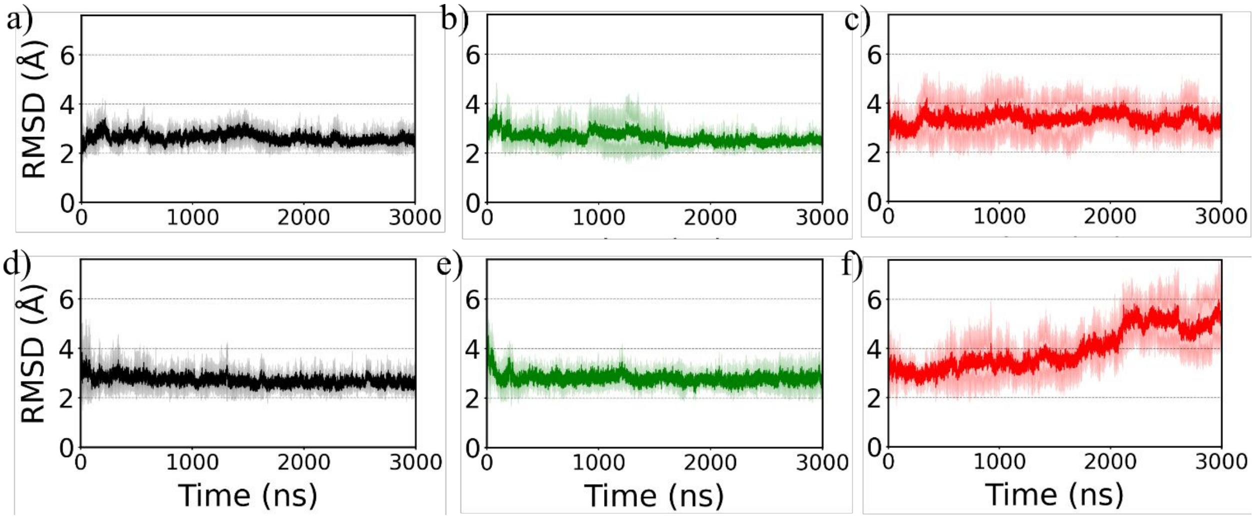

Figure S9: Time evolution of the backbone RMSD for the (a) WT (black), (b) L27A (green), and (c) L28A (red) systems obtained from CHARMM36m simulations. Data are averaged over three independent trajectories per system, with the shaded region representing the standard deviation. For comparison, the corresponding results from CHARMM36mW simulations are shown in (d), (e), and (f), respectively.

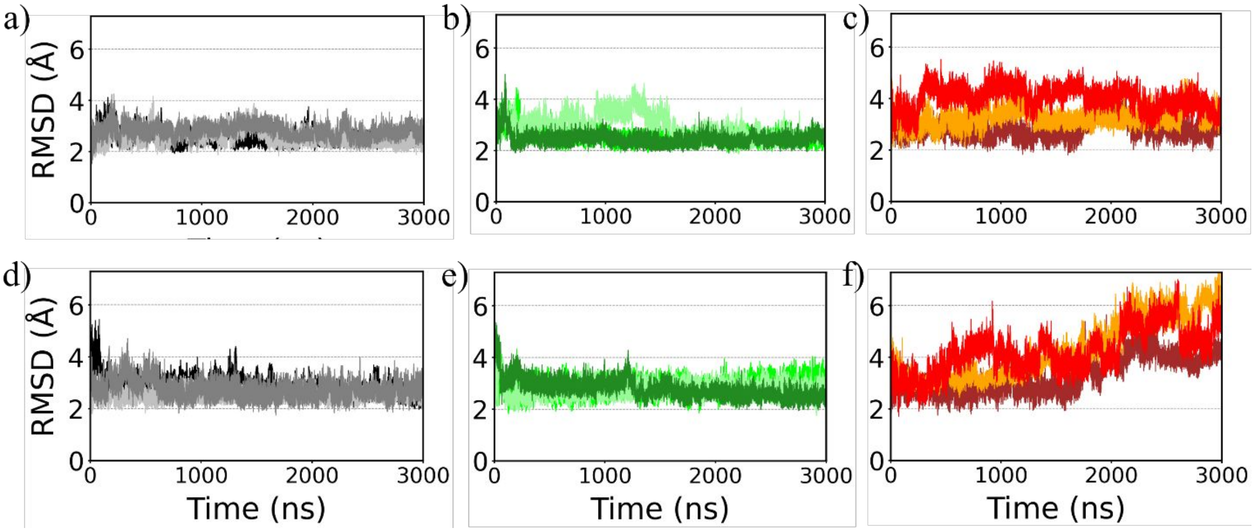

Figure S10: Time evolution of backbone RMSD for three independent trajectories of the (a) WT, (b) L27A, and (c) L28A systems obtained using CHARMM36m simulations. For comparison, the corresponding results from CHARMM36mW simulations are shown in (d), (e), and (f), respectively.

Table S6: Mean values of RMSD, R_g, hydrophobic SASA, and H-bond count obtained from CHARMM36m simulations. Data are averaged over the final 1.0 μs of three independent trajectories per system. Standard deviations are provided within braces. Corresponding results from CHARMM36mW simulations are also included for comparison.

System	Force field	RMSD (Å)	R_g (Å)	Hydrophobic SASA (Å^2)	H-bond counts
WT	CHARMM36m	2.5(±0.2)	11.8(±0.1)	1534.2(±84.2)	39.8(±4.8)
	CHARMM36mW	2.6(±0.2)	11.8(±0.1)	1593.0(±93.3)	38.0(±4.9)
L27A	CHARMM36m	2.5(±0.2)	11.7(±0.1)	1506.6(±63.7)	41.2(±4.2)
	CHARMM36mW	2.7(±0.3)	11.9(±0.1)	1643.2(±136.4)	38.1(±4.5)
L28A	CHARMM36m	3.3(±0.5)	12.0(±0.1)	1719.6(±95.8)	33.7(±4.5)
	CHARMM36mW	5.0(±0.9)	12.3(±0.2)	1825.6(±157.9)	31.8(±4.8)

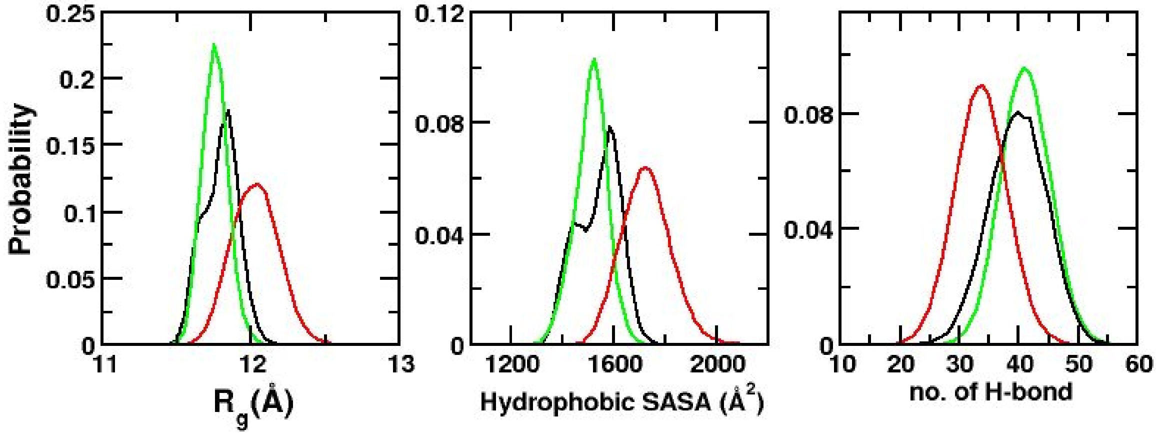

Figure S11: Normalized distributions of radius of gyration (R_g), solvent-accessible surface area of hydrophobic residues (Hydrophobic SASA), and the number of hydrogen bonds (H-bond) for the WT (black), L27A (green), and L28A (red) systems, obtained from CHARMM36m simulations. Data are averaged over the final 1.0 μs of three independent trajectories per system.

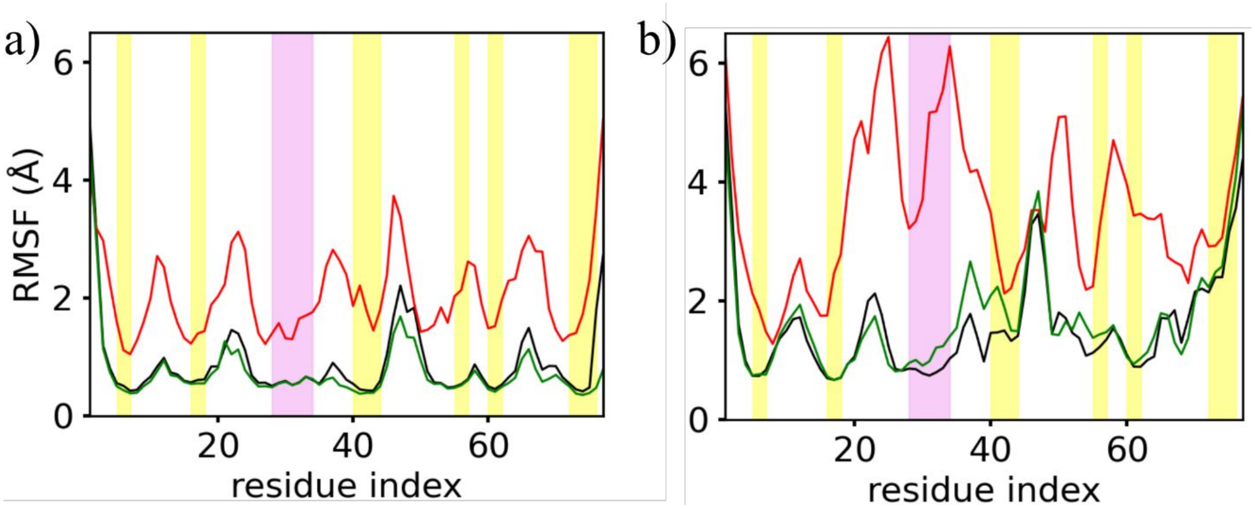

Figure S12: (a) Residue-wise backbone RMSF for WT (black), L27A (green), and L28A (red) systems from CHARMM36m simulations. Data are averaged over the final 1.0 μs of three independent trajectories per system. β-sheet and α-helix regions in the native structure are colored in yellow and violet, respectively. Corresponding results from CHARMM36mW simulations are presented in (b) for comparison.

Table S7: Total percentage of α-helix and β-sheet content measured from CHARMM36m simulations. Data are averaged over the final 1.0 μs of three independent trajectories per system. Corresponding results from CHARMM36mW simulations are also included for comparison.

System	Force field	α-helix	β-sheet
WT	CHARMM36m	9.0	35.0
	CHARMM36mW	9.0	36.0
L27A	CHARMM36m	9.0	39.0
	CHARMM36mW	8.0	33.0
L28A	CHARMM36m	7.0	28.0
	CHARMM36mW	5.0	24.0

Table S8: Residue-wise β-sheet (%) obtained from CHARMM36m simulations. Data are averaged over the final 1.0 μs of three independent trajectories per system. β-strand regions present in the native structure are indicated, with the corresponding β-sheet (%) from the simulations shown in bold. Mutation sites are shown in red, and residues with β-sheet occurrence over 10% during the simulation, along with their corresponding β-sheet (%), are shown in green.

	**residue**	**WT**	**L27A**	**L28A**
	1 MET	0.0	0.0	0.0
	2 SER	0.7	0.4	0.2
	3 GLU	13.0	19.2	0.3
	4 TYR	69.5	94.7	58.3
**β1**	**5 ILE**	**99.7**	**100.0**	**63.3**
**β1**	**6 ARG**	**99.9**	**100.0**	**82.7**
**β1**	**7 VAL**	**100.0**	**100.0**	**97.7**
	8 THR	100.0	100.0	87.4
	9 GLU	0.1	0.0	10.6
	10 ASP	0.1	0.0	0.0
	11 GLU	0.0	0.0	0.0
	12 ASN	0.0	0.0	0.6
	13 ASP	0.0	0.0	0.7
	14 GLU	0.0	0.0	0.7
	15 PRO	0.3	0.1	1.7
**β2**	**16 ILE**	**99.9**	**99.9**	**80.0**
**β2**	**17 GLU**	**99.9**	**99.9**	**79.8**
**β2**	**18 ILE**	**99.7**	**100.0**	**62.7**
	19 PRO	69.5	94.7	58.1
	20 SER	13.2	19.2	1.0
	21 GLU	0.4	0.1	0.8
	22 ASP	0.0	0.0	0.0
	23 ASP	0.0	0.0	0.0
	24 GLY	0.0	0.0	0.0
	25 THR	58.3	97.8	12.7
	26 VAL	99.6	99.9	55.9
	27 LEU	99.5	99.8	54.6
	28 LEU	0.0	0.0	0.0

	29 SER	0.0	0.0	0.0
	30 THR	0.0	0.0	0.0
	31 VAL	0.0	0.0	0.0
	32 THR	0.0	0.0	0.0
	33 ALA	0.0	0.0	0.0
	34 GLN	0.0	0.0	0.0
	35 PHE	0.0	0.0	0.0
	36 PRO	0.0	0.0	0.0
	37 GLY	0.0	0.0	0.0
	38 ALA	0.0	0.0	1.2
	39 CYS	0.0	0.0	17.8
**β3**	**40 GLY**	**38.8**	**58.7**	**30.4**
**β3**	**41 LEU**	**67.2**	**99.8**	**63.9**
**β3**	**42 ARG**	**99.8**	**100.0**	**97.8**
**β3**	**43 TYR**	**99.9**	**100.0**	**99.4**
**β3**	**44 ARG**	**86.6**	**95.8**	**97.2**
	45 ASN	8.3	11.2	22.6
	46 PRO	0.0	0.0	0.0
	47 VAL	0.0	0.0	0.0
	48 SER	0.4	0.7	0.3
	49 GLN	0.4	0.7	0.4
	50 CYS	8.3	11.2	22.6
	51 MET	86.6	95.8	77.0
	52 ARG	99.9	100.0	81.7
	53 GLY	99.6	99.6	72.3
	54 VAL	1.5	1.1	14.0
**β4**	**55 ARG**	**61.1**	**68.4**	**35.3**
**β4**	**56 LEU**	**99.1**	**99.5**	**39.2**
**β4**	**57 VAL**	**97.7**	**98.5**	**38.7**
	58 GLU	0.0	0.0	0.0
	59 GLY	0.0	0.0	0.0
**β5**	**60 ILE**	**100.0**	**100.0**	**65.1**
**β5**	**61 LEU**	**100.0**	**100.0**	**66.3**
**β5**	**62 HIS**	**79.2**	**98.7**	**44.7**
	63 ALA	0.0	0.0	0.0
	64 PRO	0.0	0.0	0.0
	65 ASP	0.0	0.0	0.0
	66 ALA	0.0	0.0	0.0
	67 GLY	0.0	0.0	0.1
	68 TRP	0.0	0.0	0.2
	69 GLY	0.0	0.0	0.8
	70 ASN	0.0	0.0	49.6
	71 LEU	0.0	0.0	98.5
**β6**	**72 VAL**	**63.2**	**81.6**	**99.5**
**β6**	**73 TYR**	**100.0**	**100.0**	**81.5**
**β6**	**74 VAL**	**100.0**	**100.0**	**31.5**
**β6**	**75 VAL**	**66.6**	**99.8**	**17.8**
**β6**	**76 ASN**	**38.9**	**58.7**	**1.2**
	77 TYR	0.0	0.0	0.0

Table S9: Residue-wise α-helix (%) obtained from CHARMM36m simulations. Data are averaged over the final 1.0 μs of three independent trajectories per system. α-helix regions present in the native structure are indicated, with the corresponding α-helix (%) from the simulations shown in bold. Mutation sites are highlighted in red.

	residue	WT	L27A	L28A
	1MET	0.0	0.0	0.0
	2 SER	0.0	0.0	0.5
	3 GLU	0.0	0.0	0.5
	4 TYR	0.0	0.0	0.5
	5 ILE	0.0	0.0	0.5
	6 ARG	0.0	0.0	0.0
	7 VAL	0.0	0.0	0.0
	8 THR	0.0	0.0	0.0
	9 GLU	0.0	0.0	0.0
	10 ASP	0.0	0.0	0.0
	11 GLU	0.0	0.0	0.0
	12 ASN	0.0	0.0	0.0
	13 ASP	0.0	0.0	0.0
	14 GLU	0.0	0.0	0.0
	15 PRO	0.0	0.0	0.0
	16 ILE	0.0	0.0	0.0
	17 GLU	0.0	0.0	0.0
	18 ILE	0.0	0.0	0.0
	19 PRO	0.0	0.0	0.0
	20 SER	0.0	0.0	0.0
	21 GLU	0.1	0.0	0.2
	22 ASP	0.1	0.0	0.2
	23 ASP	0.1	0.0	0.2
	24 GLY	0.1	0.0	0.2
	25 THR	0.0	0.0	0.0
	26 VAL	0.0	0.0	0.0
**α-helix**	**27 LEU**	**0.0**	**0.0**	**0.0**
**α-helix**	**28 LEU**	**99.8**	**99.8**	**93.8**
**α-helix**	**29 SER**	**100.0**	**100.0**	**94.8**
**α-helix**	**30 THR**	**100.0**	**100.0**	**95.0**
**α-helix**	**31 VAL**	**100.0**	**100.0**	**95.2**
**α-helix**	**32 THR**	**98.2**	**97.8**	**78.7**
**α-helix**	**33 ALA**	**86.2**	**82.9**	**58.5**
**α-helix**	**34 GLN**	**86.0**	**82.6**	**52.9**
**α-helix**	**35 PHE**	**0.0**	**0.0**	**0.0**
	36 PRO	0.0	0.0	0.0
	37 GLY	0.0	0.0	0.0
	38 ALA	0.0	0.0	0.0
	39 CYS	0.0	0.0	0.0
	40 GLY	0.0	0.0	0.0
	41 LEU	0.0	0.0	0.0
	42 ARG	0.0	0.0	0.0
	43 TYR	0.0	0.0	0.0
	44 ARG	0.0	0.0	0.0
	45 ASN	0.0	0.0	0.0
	46 PRO	0.0	0.0	0.0

	47 VAL	0.0	0.0	0.0
	48 SER	0.0	0.0	0.0
	49 GLN	0.0	0.0	0.0
	50 CYS	0.0	0.0	0.0
	51 MET	0.0	0.0	0.0
	52 ARG	0.0	0.0	0.0
	53 GLY	0.0	0.0	0.0
	54 VAL	0.0	0.0	0.0
	55ARG	0.0	0.0	0.0
	56 LEU	0.0	0.0	0.0
	57 VAL	0.0	0.0	0.0
	58 GLU	0.0	0.0	0.0
	59 GLY	0.0	0.0	0.0
	60 ILE	0.0	0.0	0.0
	61 LEU	0.0	0.0	0.0
	62 HIS	0.0	0.0	0.0
	63 ALA	0.0	0.0	0.0
	64 PRO	0.0	0.0	0.0
	65 ASP	0.0	0.0	0.0
	66 ALA	0.0	0.0	0.0
	67 GLY	0.0	0.0	0.0
	68 TRP	0.0	0.0	0.0
	69 GLY	0.0	0.0	0.0
	70 ASN	0.0	0.0	0.0
	71 LEU	0.0	0.0	0.0
	72 VAL	0.0	0.0	0.0
	73 TYR	0.0	0.0	0.0
	74 VAL	0.0	0.0	0.0
	75 VAL	0.0	0.0	0.0
	76 ASN	0.0	0.0	0.0
	77 TYR	0.0	0.0	0.0

Table S10: Results obtained from the MUpro, SAAFEC and DynaMut servers for the L27A and L28A mutants. ENCoM is also based on DynaMut, but additionally incorporates vibrational entropy derived from normal mode analysis.

mutant	ΔΔG (kcal/mol)			
	MUpro	SAAFEC	DynaMut	ENCoM
L27A	-1.28	-1.86	-0.25	-0.11
L28A	-2.56	-2.66	-0.92	-0.36
